# Mesenchymal stem cells- derived exosomes inhibit the expression of Aquaporin-5 and EGFR in HCT-116 human colorectal carcinoma cell line

**DOI:** 10.1186/s12860-022-00439-0

**Published:** 2022-09-16

**Authors:** Amir Hossein Mansourabadi, Azin Aghamajidi, Fatemeh Faraji, Shirin Taghizadeh, Leila Mohamed Khosroshahi, Mona Bahramkiya, Maryam Azimi

**Affiliations:** 1 Department of Immunology, School of Medicine, Tehran University of Medical Sciences, Tehran, Iran; 2 Immunogenetics Research Network (IgReN), Universal Scientific Education and Research Network (USERN), Tehran, Iran; 3 Systematic Review and Meta-Analysis Expert Group (SRMEG), Universal Scientific Education and Research Network (USERN), Tehran, Iran; 4 Department of Immunology, School of Medicine, Iran University of Medical Sciences, Tehran, Iran; 5 Immunology Research Center, Institute of Immunology and Infectious Diseases, Iran University of Medical Sciences, Hemmat highway, Tehran, Iran; 6 Department of Pharmaceutical and Medicinal Chemistry, Institute of Pharmacy, University of Bonn, 53113 Bonn, Germany

**Keywords:** Aquaporin 5, Exosome, Mesenchymal stem cell, HCT116

## Abstract

**Background:**

Aquaporins are channel proteins, form pores in the membrane of biological cells to facilitate the transcellular and transepithelial water movement. The role of Aquaporins in carcinogenesis has become an area of interest. In this study, we aimed to investigate the effects of adipose-derived mesenchymal stem cells secreted exosomes on the expression of aquaporin 5 and EGFR genes in the HCT-116 tumor cell line.

**Methods and results:**

Surface antigenic profile of Ad-MSCs was evaluated using specific markers. Exosomes were purified from the Ad-MSc supernatant while the quality and the shape of isolated exosomes were assessed by western blot and transmission electron microscopy (TEM) respectively. HCT-116 cells were co-cultured with MSC-conditioned medium (MSC-CM) and/or with 100 μg/ml of MSC-derived exosomes for 48 h and. Real-time PCR was carried out to determine the expression of aquaporin5 and EGFR in HCT-116. Relative expression levels were calculated using the 2^-ΔΔct^ method.

Our result showed that AQP5 and EGFR mRNA levels were significantly reduced in CM and/or exosomes treated HCT116 compare to the control group (*P*-value < 0.05).

**Conclusion:**

The current study showed that MSC derived exosomes could inhibit expression of two important molecules involved in tumor progression. Hence it seems MSCs-derived exosomes may hold a hopeful future as drug delivery vehicles which need the furtherer investigation.

## Introduction

Colorectal cancer (CRC) is the second most lethal cancer in women and men worldwide [1]; which it is expected that the universal burden of CRC increases by 60% to more than 2.2 million new cases and 1.1 million deaths by 2030 [2]. Studies have demonstrated genetics, age, Smoking, obesity, unhealthy diet, and physical inactivity, contribute to CRC development [3]. The common treatment of CRC is surgery and adjuvant chemotherapy to the complete removal of the tumor. However, most CRCs cases are diagnosed at an advanced stage with metastases to other organs i.e. liver which results in difficulties in surgical intervention and consequent tumor-related deaths. In addition, 20 to 50% of patients eventually experience recurrence of the tumor after surgery and subsequent chemotherapy [4]. Hence, identifying the underlying molecular mechanisms of metastasis of colorectal cancer seems vital for colorectal cancer therapy.

Aquaporins (AQPs) are the family of 13 transmembrane water channel proteins (AQP 0–12) which facilitate the transcellular transport of water, glycerol, hydrogen peroxide, and other small water-soluble material [5]. AQPs are widely distributed in various tissues throughout the body and have an indispensable effect on cell volume and water homeostasis [6].

Several studies have reported that dysregulation of AQPs plays a critical role in several pathophysiological conditions including malignancy. Indeed, expression is positively correlated with tumor types, grades, proliferation, migration, angiogenesis, etc., in various tumors like colon, ovarian, brain lung, and pancreatic cancers [7–11]. Therefore, AQPs have become an interesting concept in cancer research especially as diagnostic and therapeutic targets in anticancer treatment.

**Fig. 1:**
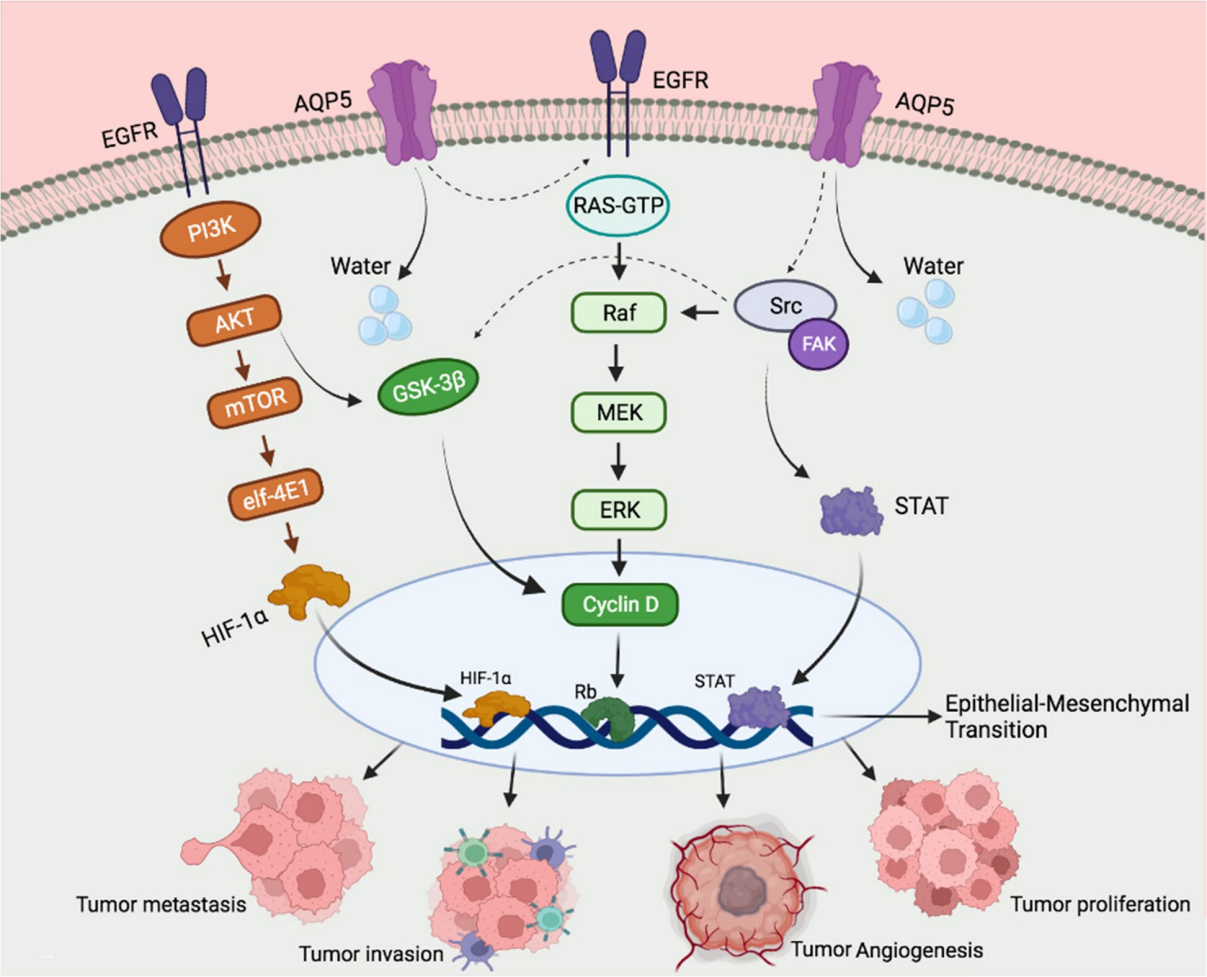
AQP5 and EGFR interaction in tumor biology. AQP5 induces extracellular receptor kinase (ERK1/2) pathway activation via activation of the epidermal growth factor receptor (EGFR), leading to facilitating tumor proliferation and metastasis. Besides, AQP5 is phosphorylated followed by binding to the SH3 domain of Src to promote epidermal mesenchymal transition (EMT) activity in tumor cells

AQP1, AQP3, AQP5, and AQP9 are associated with colorectal cancers [12]; among them, it has been indicated that AQP5 promotes the proliferation and metastasis of CRC [12–14]; moreover, elevated expression of AQP5 in CRC tissue is associated with a poor prognosis of colorectal cancer [15, 16]. AQP5 and its upstream and downstream signaling pathways play a significant role in tumor proliferation, invasion, and metastases (Fig. 1). Interestingly, it has been shown that AQP5 activates the Epidermal Growth Factor Receptor (EGFR) and Extracellular signal-Regulated Kinases 1/2 (ERK1/2) pathway, resulting in tumor cell proliferation and migration [17, 18] Briefly, up-regulating AQP5 in tumor cells stimulates EGFR that in turn trigger the RAS/MAPK as well as phosphatidylinositol-3- kinase (PI3K)/AKT signal pathways; PI3K activates AKT which blocks caspase-9, finally hindering apoptosis in AQP5 expressing cancer cells [18, 19].

Yang et al. has shown that in tumor cells expressing high level of AQP5, EGFR phosphorylation was enhanced, and the ERK and MAPK signaling pathways were activated; conversely, the activity of the EGFR/ERK/p38 MAPK pathway has been reduced following AQP5 gene silencing [20].

In general, due to the ineffectiveness of surgery and chemotherapy as well as the high invasion potential of tumor cells, new medicine approaches has been applied to mesenchymal stem cell (MSCs) therapy to treat various malignancy condition including CRC. These multipotent MSCs are important regulators of immune system; they have a long-term self-renewal capacity and multidirectional differentiation [21].

Moreover, MSCs can migrate to an inflammatory or tumor site and display profound immune-modulatory [22, 23]. Exosome releasing is one of the fundamental immunomodulatory mechanisms of MSCs. Exosomes are Nano-vesicles (~ 30–200 nm in diameter) membrane-bound which are secreted from various cell types [23]. These small vesicles transport proteins, lipids, nucleic acids, and other substances between cells; so, they are known as intercellular communication vehicles [23].

Although, a number of studies have shown that mesenchymal stem cells derived-exosomes showed a potential for cancer treatment [24, 25]; to date, there have been limited data regarding the role of MSCs-derived exosomes on colorectal cancer development. Therefore, the current study investigated the effect of MSCs-derived exosomes on aquaporin 5 and EGFR expression in human colon carcinoma cell lines.

## Materials and methods

### Cell culture

Adipose-derived mesenchymal stem cells (Ad-MSCs) were supplied by the GenIran research & education center (Tehran, Iran). Colorectal cancer cell line (HCT-116) was obtained from Pasture Institute (Tehran, Iran); all cells were cultured in DMEM/F12 medium supplemented with 10% fetal bovine serum (FBS), 100 IU/ml penicillin/ 100 Ug/ml streptomycin (P/S) and 1% L-glutamine.

### Characterization of ad-MSCs

The surface antigenic profile of Ad-MSCs was evaluated using a flow cytometer FACSCalibur (BD Biosciences, USA); to phenotyping of Ad-MSCs, four antibodies against human-MSC Markers were used, including, CD90, CD73, CD45, and CD34 (all from eBioscience).

### Preparation of ad-MSC-conditioned media and isolation of exosomes

After receiving of Ad-MSCs, the cell medium was changed every other day till reaching about 70% confluence. Then, every 2 days, the medium of MSCs from passage 3, was replaced with the medium containing lower FBS. MSCs were gradually adapted to the FBS-free medium. Finally, after 4 h, FBS-free supernatants were collected and filtered by 0.22 μm filters.

Exosomes were purified from the Ad-MSc supernatant using an Exosome Isolation kit (EXOCIB, Iran) according to the manufacturer’s protocol. Briefly, the collected supernatant was centrifuged at 3000 RPM for 20 min to remove cellular debris. The supernatant was transferred to a new 1.5 ml tube and the pre-heated reagent-A was added as a 1:5 ratio. Following the incubation overnight at 4 °C, the mixture was centrifugated at 3000 RPM for 45 min and the supernatant was discarded. The pellet of the exosomes was resuspended with reagent-B and the total protein concentration was measured for exosome quantification using the Bradford method.

### Characterization of MSC-exosomes

The quality of isolated exosomes was assessed by western blot using a primary antibody (mouse anti-CD63 antibody, the concentration of 100 μg/mL, (Abcam, USA) and secondary HRP conjugated anti-mouse IgG, the concentration of 200 μg/mL (Santa Cruz, USA). In addition, transmission electron microscopy (TEM) (ZeissEM10C) was done to confirm the shape of the isolated exosomes.

The purified exosomes were also labeled with the anti-human antibodies, including anti-CD81 and anti-CD63 antibodies, for flow cytometric analysis (both antibodies were purchased from eBioscience). The analysis was carried out, using a FACSCalibur flow cytometer. To analyze the size distribution of the exosomes, they were diluted in PBS and Tween-20. The size of them was measured using dynamic light scattering (DLS) Zetasizer Nano ZS (Malvern Instruments, UK).

### HCT-116 colon cancer cell lines co-culture

HCT-116 colon cancer cell lines (10^6^ cells per well) were seeded into 6 -well plates. HCT-116 cells were cultured alone (no treated), with MSC-conditioned medium (MSC-CM) in 1:1 ratio or with 100 μg/ml of MSC-derived exosomes respectively for 48 h. All experiments were performed in triplicate.

### RNA extraction and reverse transcription

Total RNA was extracted from cells using the AnaCell Super RNA extraction kit (Ana Cell tec., Iran) according to the manufacturer’s instructions. The concentration and purity of RNA were analyzed by measuring absorbance at 260/280 nm by a Nanodrop spectrophotometer (Thermo Fisher Scientific). Reverse transcription was performed using the cDNA Synthesis Kit (Ana Cell tec, Iran) according to the manufacturer’s protocols.

### mRNA quantification

Aquaporin-5 and EGFR genes specific primers were designed by NCBI Primer Blast Software. Real-time PCR (RT-PCR) was carried out using Amplicon SYBR Green PCR Kit (Amplicon, Denmark). The GAPDH gene was used as an internal control for normalization. All quantitative PCR measurements were performed using Rotor-gene Q thermal cycler (Qiagen, Q, Germany). Reactions were carried out in 20 μL final volumes, including 10 μL of SYBR Green PCR Master Mix (Amplicon, Denmark), 0.5 μL of both forward and reverse primer, 1 μL of undiluted cDNA, and 8 μL of nuclease-free water (CinnaGen, Tehran, Iran) The threshold cycles were normalized to GAPDH and the relative expression levels were calculated using the 2^-ΔΔct^ method.

### Statistical analysis

Statistical analysis was performed using Prism (GraphPad) software. Kruskal–Wallis test was used for the analysis of data. Data are presented as Mean ± SEM and *P* values < 0.05 were considered significant.

## Results

### Characterization of ad-MSCs

**Fig. 2:**
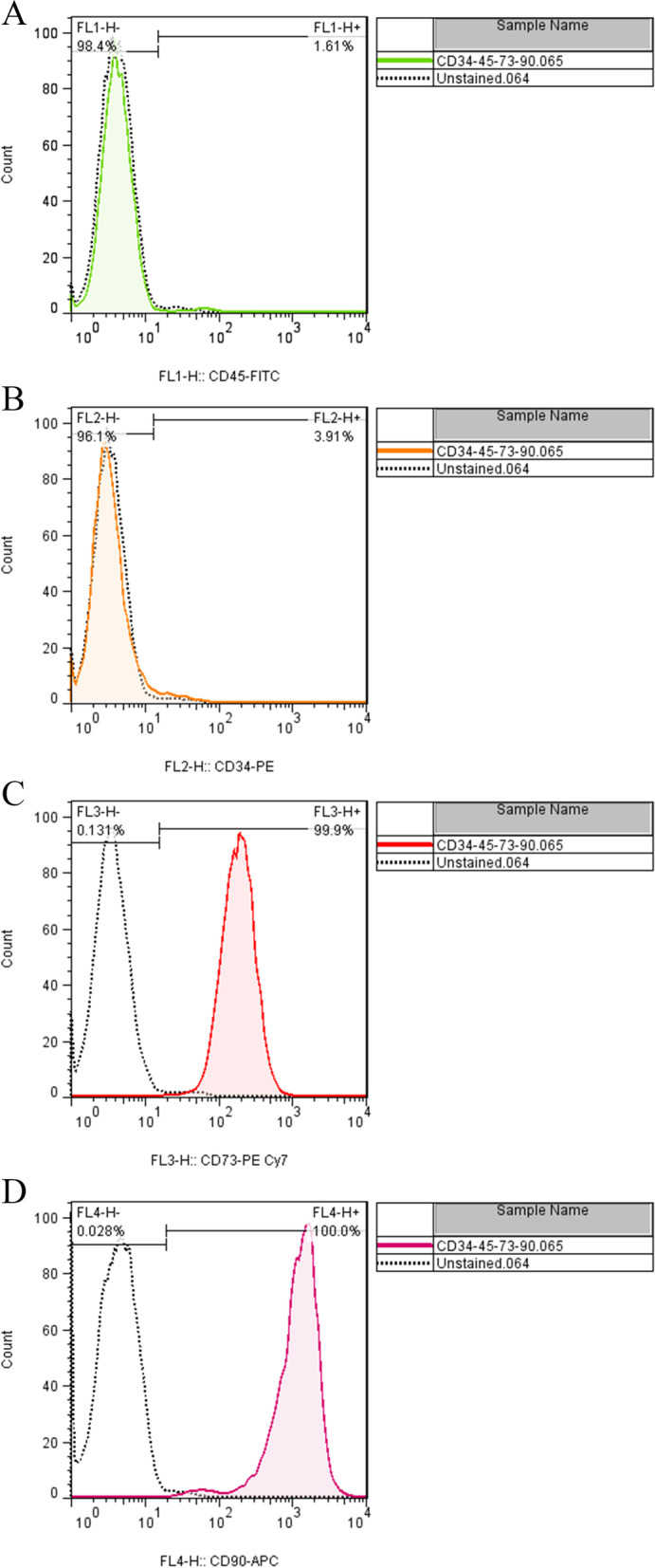
Flowcytometric immunophenotyping for surface markers of Ad-MSCs. The majority of MSCs were positive for surface markers including CD73 (**A**), CD90 (**B**), and negative for CD45 (**C**), and CD34 (**D**)

Morphological assessment by light microscopy showed that the MSCs grew as adherent cells with fibroblast-like shape morphology. Surface antigen immunophenotyping demonstrated that adipose tissue-derived MSCs (Ad-MSc) positively express CD73 and CD90 while they were negative for CD34and CD45 markers (Fig. 2A-D).

### Characterization of MSC-derived exosomes

**Fig. 3:**
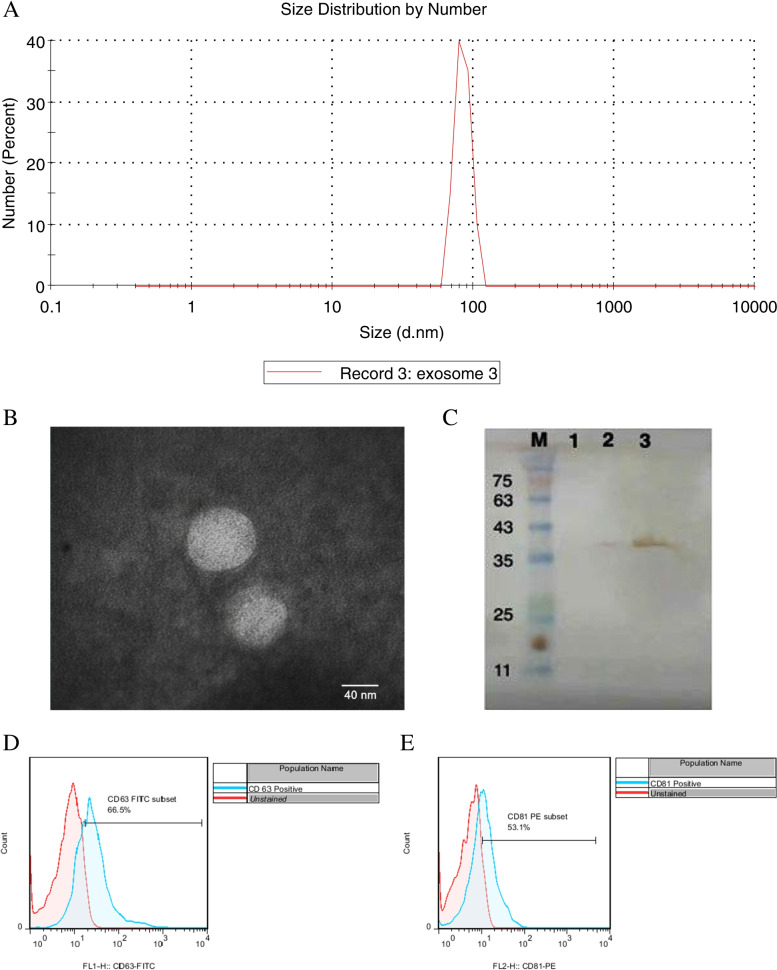
Characterization of the purified exosome. MSC-derived exosomes were dispersed in phosphate buffer saline and then measured at 30 μg/mL concentration (the figure shows a representative line plot of one sample. All samples were assessed in duplicate). Exosome size distribution by dynamic light scattering (DLS) is shown (**A**). Morphology of UC-MSC-derived exosomes under a transmission electron microscope (TEM, 20000 x magnification) (**B**). western blot analysis of isolated MSCs-derived exosomes. Lane 2 and 3 are shown two different concentrations of 15.0 and 150 μg/ml, respectively. Lane 1 is a PBS, as a negative control (**C**). CD63 and CD8 surface marker expression of isolated exosomes by flow cytometry (**D**)

To confirm the shape and size distribution of the purified exosomes, TEM and DLS were performed respectively (Fig. 3A, B). As shown in Fig. 3A, the mean size of over 90% of exosomes was ~ 100 nm in diameter. Based on western blot and flow cytometry analyses, the isolated exosomes were positive for exosome-specific markers CD63 and CD81 (Fig. 3C-E).

### Down-regulation of Aquaporin-5 in exosome treated cells

**Fig. 4:**
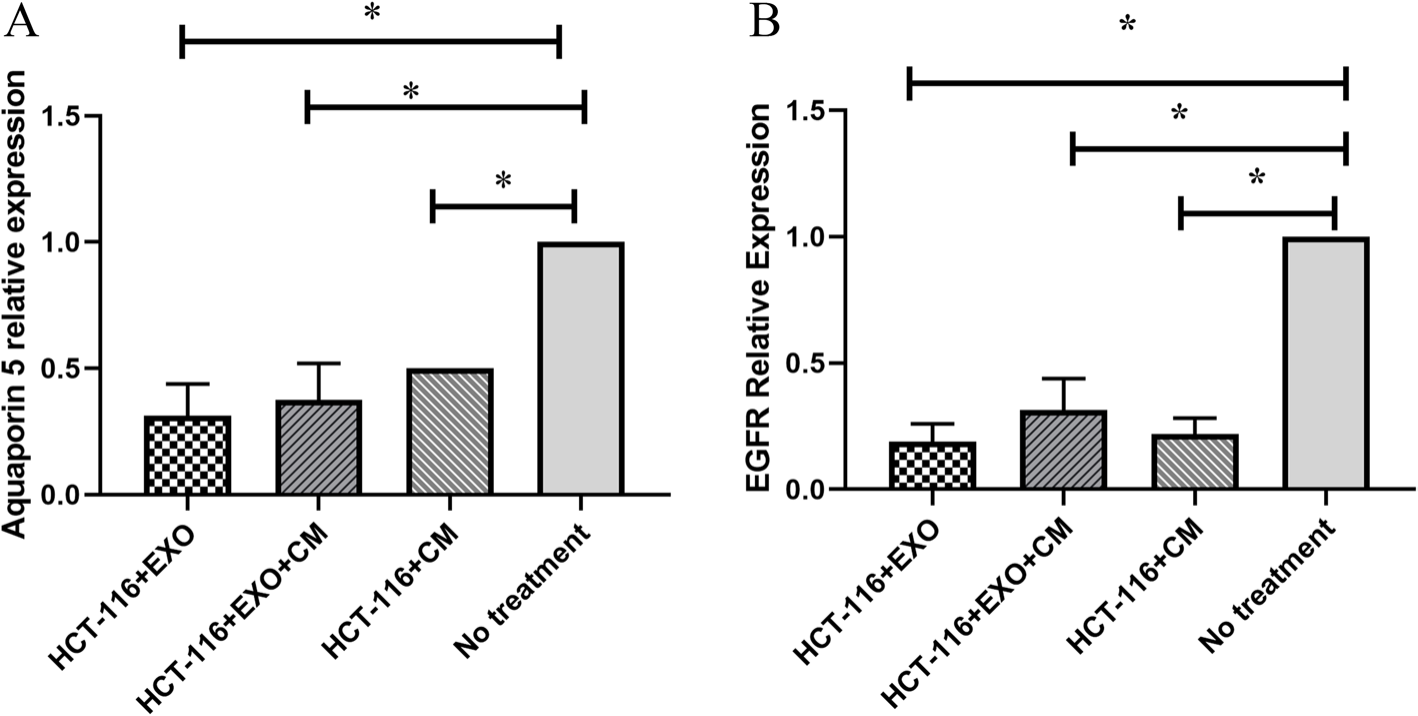
Expression of AQ5 and EGFR in treated groups. HCT-116 cells were co-cultured with MSC-CM and/or MSC-derived exosomes for 48 h and the expression levels of AQP5 and EGFR were determined by real-time PCR. AQP5 (**A**) and EGFR (**B**) mRNA expression levels were significantly reduced in all treated groups compared with the untreated group. The Values shown are mean ± SEM and *P* values < 0.05 were considered significant

In order to assess the expression of AQP5 and EGFR in the presence or absence of MSC-CM and/or MSC-Exosomes; ~ 10^6^ HCT-116 cells were co-cultured with MSC- conditioned medium (MSC-CM) and/or 100 μg/ml isolated CD63^+^ exosomes. The result showed that AQP5 and EGFR mRNA levels were significantly reduced in CM and/or exosomes treated HCT116 relative to the No treated cells (Fig. 4A and B, p < 0.05).

## Discussion

Generally, colorectal cancer is one of the most lethal human malignancies [1]. High rate cancer cells metastasis plays a significant role in the CRC treatment failure [26, 27]; therefore, to design effective therapeutic approaches, there is a vital necessity to reveal critical pathological target molecules.

In this research project, we evaluated the immunomodulatory effects of MSC-derived exosomes, as well as MSC’s conditioned media, on AQP5 and EGFR gene expression in a highly invasive colorectal cancer cell line “HCT-116”. For this purpose, we co-cultured MSC- conditioned medium (MSC-CM) and isolated CD63 + exosomes (~ 100 nm in diameter) with HCT-116 cell line. We indicated that compared to no-treated cells, both MSC-derived exosomes and MSC-CM reduced expression of AQP5 and EGFR in HCT-116.

AQPs are a family of transmembrane proteins, which control the transverse transportation of water-electrolytes [24]. Matsuzaki et al. showed that AQP5 is located in the intercellular secretory tubules of secreting cells of salivary glands, pyloric glands, and duodenal glands with a significant role in water transportation [28].

Consistent with our data, emerging evidence has demonstrated an increase in AQP expression within various tumors, especially invasive tumors, such as colorectal cancer [29]. It also has been shown that AQPs are involved in tumor development including tumor proliferation, tumor cell migration, and tumor angiogenesis [8, 30]. Specifically, AQP5 has been shown to be associated with the development especially metastasis of CRC [15, 31], Thus, silencing of AQPs may act as a novel class of anti-tumor agents.

In 2017 Chen et al. demonstrated that overexpression of AQP5 promoted the mesenchymal-like phenotype and EMT process in the high metastatic colorectal cancer cell lines (i.e. HCT-116, SW480); while, inhibition of AQP5 resulted in the inhibition of EMT in these cell lines [31]. Moreover, it was shown that up-regulation of AQP5 in certain tumors activates EGFR followed by the activation of the ERK1/2 pathway which leads to proliferation and metastasis potential of cancer cells [18, 32]. In 2008, Kang et al. showed that overexpression of human AQP5 increased phosphorylation of ERK1/2 and proliferation in HCT116 colon cancer cells; additionally, AQP5 silencing led to reduced cell proliferation and inhibition of ERK phosphorylation [33].

Recently, identifying the mesenchymal stem cell roles in tumor development has attracted extensive research attention. Increasing evidence has revealed that MSCs show a variety of biological functions such as immune regulation, tissue damage repair, and therapeutic effects on tumors like CRC [34–36]. Studies indicated that under certain treatment conditions, MSCs can inhibit the progression of CRC [37, 38]. M. Nasuno and colleagues indicated that Bone marrow derives MSCs inhibit the Azoxymethane-Induced colon tumor Initiation [39]. Additionally, In 2021, Ruohang et al. showed that MSCs therapy could inhibit tumor progression and chronic inflammation in animal models of Colitis-Associated Colorectal Cancer [40].

Among the paracrine manner of MSCs, exosome releasing has been an attractive investigation area. Like the maternal cells, MSC-derived exosomes present cell-specific tropism, desirable biocompatibility, and low immunogenicity which provide them a versatile therapeutics carrier to the site of action [38, 41, 42]. It seems the effect of MSC-derived exosomes on tumor progression is a two-edged sword that has been extensively investigated. Several studies suggest that MSCs derived exosomes by transferring tumor-supportive material prompt tumorigenesis, while other studies indicated that MSC-derived exosomes play a significant role in tumor suppression [23]. The exact mechanism of MSCs-derived exosomes on aquaporin 5 and EGFR expression has not been cleared. In current one of the probable mechanisms is that MSC-derived exosomes transferred inhibitor mediators to colorectal cancer cells which suppressed tumor progression. to colorectal cancer cells which suppressed tumor progression. Interestingly, in 2013, Wu et al. demonstrated that human MSCs derived exosomes by down-regulating phosphorylation of Akt protein kinase and up-regulating cleaved caspase-3 suppressed the development of bladder carcinoma cells [43]. Correspondingly, it was shown that via delivery of miR-145 “tumor suppressor microRNA” adipose -derived exosomes significantly inhibited cancer proliferation and induce apoptosis in these cells via activating the caspase-3/7 mediated apoptosis pathway and suppressing the anti-apoptotic activity of Bcl-xL [44]. However, the role of exosomes and membrane-associated receptors, particularly EGFR as mediators of cell proliferation and invasion in cancer progression remains unexplored. EGFR is mostly overexpressed and has been correlated with aggressive forms of tumor cells. In cancer cells, hyperactivity of EGFR is linked with androgen independence and metastasis of prostate cancer cells [45]. Since exosomes are involved in cell-cell communication that alters the phenotype of the recipient/target cells [46, 47], Exosomal-EGFR expressed by various tumor types may play an important role in cancer progression. Interestingly, it was shown that MiR-146b in MSC exosomes can suppress tumor growth in culture. MiR-146b from MSC exosomes binds to EGFR mRNA, and eventually reduced the growth, migration, and invasion of cancer cells in culture [48]. The characterization of MSC exosomes in different subpopulations and meticulous content characterization of the loading must be inquired to the existence of heterogeneity in exosome contents that may modify the efficacy of the intervention on the target cells or tissue [49]. Investigation of the therapeutic application of MSC exosomes is still in the early stages and the precise functional mechanism of exosomes remains largely unclear.

Our results indicated that MSC-CM and/or MSC-derived exosomes can significantly reduce AQP5 and EGFR mRNA expression levels in all treated groups compared with the untreated group in culture. It seems that there is no significant difference between MSC-derived exosomes and MSC’s conditioned media in terms of AQP5 and EGFR mRNA expression levels. While there are only a few studies that directly compare MSC-derived exosomes and MSC’s conditioned media, the overlapping effects seem to indicate that MSC-EV have a greater likelihood of impacting tumor. Although the mechanism responsible for the slight difference in effects between MSC-derived exosomes and MSC’s conditioned media is unclear, this study clarified that exosomes act against tumor development through reducing the expression of AQP5 and EGFR cell surface receptors. Our results suggest that exosomes can be used therapeutically to target EGFR-expressing tumor. However, investigating the contents of these exosomes requires more detailed studies. In the future, novel anti-cancer therapies for colorectal cancer could be developed from the findings in this study.

Collectively, as the Source of the MSC and culture condition may affect the features of the released exosomes; hence, there is a need for further research to develop a standard condition for MSC exosome isolation.

## Conclusion

In conclusion, the current study showed that mesenchymal stem cells derived exosomes could inhibit the expression of two important molecules involved in tumor progression. Hence it seems MSCs-derived may hold a hopeful future as drug delivery vehicles that need the furtherer investigation.

## Data Availability

All data generated or analyzed during this study are included in this published article.
